# Shoes and Insoles: The Influence on Motor Tasks Related to Walking Gait Variability and Stability

**DOI:** 10.3390/ijerph17124569

**Published:** 2020-06-25

**Authors:** Luca Russo, Roberto Di Capua, Benedetto Arnone, Marta Borrelli, Roberto Coppola, Fabio Esposito, Johnny Padulo

**Affiliations:** 1 Department of Biotechnological and Applied Clinical Sciences, University of L’Aquila, 67100 L’Aquila, Italy; luca83russo@gmail.com (L.R.); benedetto.arnone@univaq.it (B.A.); 2 Department of Physics “E. Pancini”, University of Naples “Federico II”, and CNR-SPIN Institute, 80126 Naples, Italy; roberto.dicapua@unina.it; 3 Department of Biomedical Sciences for Health, Università degli Studi di Milano, 20133 Milan, Italy; marta.borrelli@unimi.it (M.B.); fabio.esposito@unimi.it (F.E.); 4 Faculty of Human and Society Sciences-University of Enna “Kore”, 94100 Enna, Italy; robertogcoppola@gmail.com; 5 Faculty of Kinesiology, University of Split, 21000 Split, Croatia

**Keywords:** barefoot, electroencephalogram (EEG), foot, Lyapunov exponent, motor control

## Abstract

The rhythmic control of the lower limb muscles influences the cycle-to-cycle variability during a walking task. The benefits of insoles, commonly used to improve the walking gait, have been little studied. Therefore, the aim of this study was to assess the walking gait variability and stability on different walking conditions (without shoes, WTS, with shoes, WS, with shoes and insoles, WSI) related to brain activity. Twelve participants randomly (WTS/WS/WSI) walked on a treadmill at 4 km/h for 10 min. Kinematic analysis (i.e., footstep and gait variability), brain activation (beta wave signal), rating of perceived exertion (RPE, CR-10 scale), and time domain measures of walking variability were assessed. The maximum Lyapunov exponent (LyE) on the stride cycle period’s datasets was also calculated. Stride length and cycle calculated for all walking conditions were 61.59 ± 2.53/63.38 ± 1.43/64.09 ± 2.40 cm and 1.11 ± 0.03/1.14 ± 0.03/1.15 ± 0.04 s (F_1,10_ = 4.941/*p* = 0.01, F_1,10_ = 4.938/*p* = 0.012) for WTS, WS, WSI, respectively. Beta wave (F_1,10_ = 564.201/*p* = 0.0001) was higher in WTS compared to WS and WSI. Analysis of variance’s (ANOVA) LyE showed a F_1,10_ = 3.209/*p* = 0.056, while post hoc analysis showed a significant effect between WS and WSI with *p* = 0.023, and nonsignificant effects between WTS and WS/WSI (*p* = 0.070/0.607), respectively. Small perturbations of the foot can influence the control of gait rhythmicity by increasing the variability in a dissipative deterministic regimen.

## 1. Introduction

History shows that, although the human species is not born with shoes, their use dates back to ancient civilizations. Currently, there are many shoes on the market [1]. Some of them are suitable for specific tasks (i.e., running and sport shoes) and other kinds of shoes can be worn for every kind of use. Humans started to use shoes approximately 40,000 years ago [2]. Since that time, the shape of footwear has changed a lot, starting from simple foot protection tools to fashion gadgets [3]. These modifications, in terms of shape and function, seem to contribute to foot deformities and to abnormal function of the foot [4,5,6]. Some of the most common foot disorders are hallux valgus, plantar fasciitis, and Achilles tendinopathy. These conditions are usually treated with nonpharmacological conservative interventions, such as insoles, which are considered the first-line approach to disease management.

Walking gait is fundamental for human health and it is required to perform 10,000 steps per day [7], but how do we walk with or without shoes? Human walking in shod and barefoot conditions shows differences in terms of spatiotemporal, kinematic, and kinetic variables, as well as muscle activity [3,8,9]. Stride length is longer with shoes, and some authors suggest this could be due to a pendulum lengthening effect, which could be caused by the extra weight of the shoes giving a greater inertial load during the swing phase [10]. Despite these differences, the experimental data on long-term effects of walking in shod or barefoot conditions are not yet clear [11].

In shod condition, humans can either wear shoes only, or also use anatomical or customized insoles inside the shoes in order to fix postural problems, to treat foot pain, or just to enhance comfort [12,13]. Several authors [14,15] showed the effects of customized or prefabricated insoles on foot health, and other authors examined the effect of different types of insoles in older people or pathological subjects on gait parameters [16,17,18]. In this framework, it is interesting to study the effects of insoles on gait parameters and variability control, compared to the barefoot condition, in healthy subjects. Özmanevra et al. [19] investigated the differences in barefoot, shod, and shod plus insoles conditions in healthy subjects, comparing insoles with different materials. Chen et al. [20] investigated the differences in barefoot, shod, and shod plus insoles conditions in flat-footed subjects using only customized shoes and insoles. Each motor task requires a human to explore the immediate environment and correct the stride cycle (stride-to-stride) during the walking gait; as a consequence, stride-to-stride variability emerges as an effect of body systems to correct movement errors [21], such as the use of soles or insoles. The stride frequency variability [22] seems to reflect the requirement of the Central Pattern Generator to provide the timing activation of different lower limb muscles during the stride cycle [23]. Furthermore, the cortical activity (i.e., electroencephalogram (EEG) beta wave signals) during the walking activity can change during the new motor task as well with or without shoes or insoles. In fact, beta wave data is valued as an indicator of the attention required during walking [24]. Thus, the paradigm of the walking motor task, walking with shoes/without shoes/with shoes and insoles, could be clarified by cortical activity (EEG) and motor control, as well as gait variability. The common hypothesis is that shoes can negatively affect gait control compared to the barefoot condition, while the use of anatomical insoles inside commercial shoes could provide better comfort [25] because an increased contact surface stimulates the mechanoreceptors of the foot [26]. Therefore, the aim of this study was to assess the walking gait variability and stability of different tasks related to brain activity without shoes, with shoes and without insoles, and with shoes and insoles.

## 2. Material and Methods

### 2.1. Participants

Twelve men participated in this study (age 34.25 ± 7.81 years; body mass 72.58 ± 3.85 kg; body height 1.76 ± 0.04 m; BMI 23.52 ± 1.33 kg/m^2^). The exclusion criteria were: (i) muscular, neurological, or tendinous injuries; (ii) foot deformities or foot pain. The inclusion criteria were: male in good health and physically active. The group was homogeneous with regard to their foot health status, since none of the participants underwent any orthopedic or podiatric treatment before the study. Ethical approval was issued by the local University Ethics Committee (University of Novi Sad Ethics Committee; protocol number: 2/2019). After being fully informed of the procedures, methods, benefits, and possible risks involved in the study, each participant reviewed and signed an informed consent in accordance with the ethical standards.

### 2.2. Experimental Setting

Testing was carried out in a Sport Performance Laboratory at the temperature of 23.5 ± 0.4 °C and relative humidity of 52.4 ± 1.7% and at the same time of the day (11 am) to avoid any circadian effects [27,28]. Within this study, in order to better standardize the walking velocity, tests were performed at 0% gradient on a motorized treadmill (Run500, Technogym^TM^, Gambettola, Italy). The treadmill was calibrated before each test, according to the instructions of the manufacturer, and regularly checked after the tests [29].

**Figure 1:**
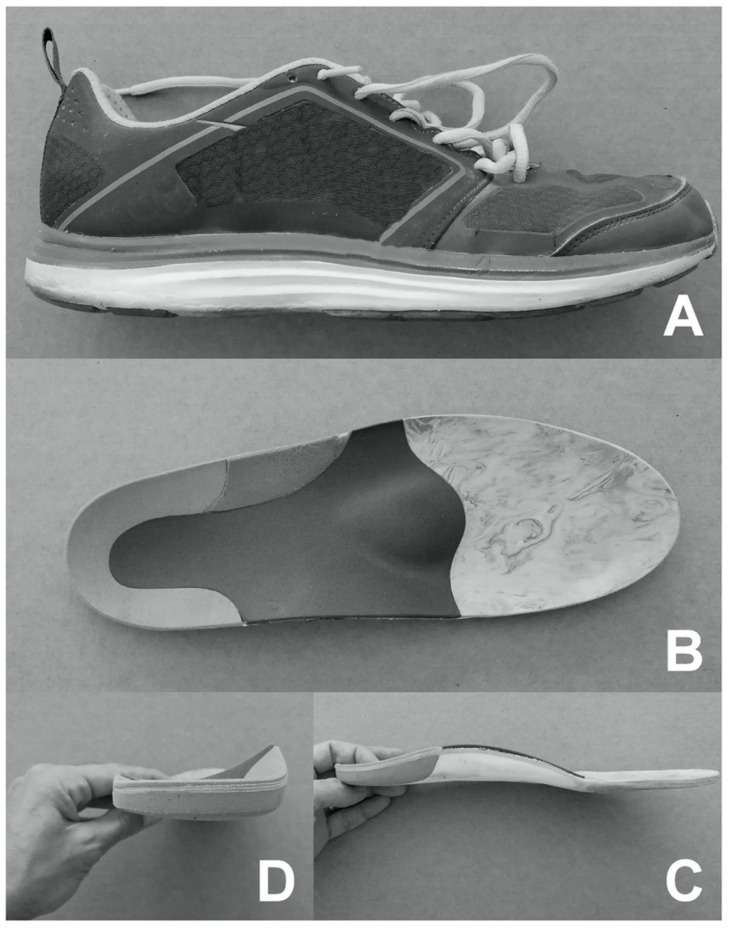
Shoes and insoles. (**A**) Lateral view of experimental shoe. The insoles were produced with an anatomical and ergonomic shape. Insoles had three-density Ethylene-Vinyl Acetate (EVA) full length, stabilization of the rear foot, support of the mid foot, and cushioning of the fore foot (**B**), physiological medial heel wedge (**C**), and physiological arch support (**D**).

All participants wore the same shoes (Figure 1A) and the same anatomical and ergonomic preformed foot insoles: a three-density full length EVA (Ethylene-Vinyl Acetate) stabilizing the rear foot, supporting the mid foot, and cushioning the fore foot (Figure 1B), with a physiological medial heel wedge (Figure 1C) and physiological arch support (Figure 1D). We used these kinds of insoles in order to choose a sample of asymptomatic subjects without flat feet or deformities; therefore, the role of the insoles is not to correct the foot, but to give anatomical and comfortable support inside the shoes, increasing the support base surface. One week before the test, the subjects had a familiarization session with the insoles.

Each subject performed 3 min walking at 3 km·h^−1^ as a standardized warm-up to familiarize themselves on the treadmill. After warm-up, each participant performed three randomized trials of 8 min walking at 4 km·h^−1^: barefoot walking (WTS), shod walking (WS), shod + insoles walking (WSI); with a 5 min standard/passive recovery between conditions. During the walking gait, each participant wore an elastic headband (MindBand, Neurosky^TM^, San Jose, CA, USA) to assess the brain activity signals. Each device was checked before/after each walking condition test, and after one week, a test was performed to assess the reliability of the measurements.

Immediately after each trial, the participants indicated their rating of perceived exertion (RPE) using the category rating-10 (CR-10) scale modified by Foster et al. [30].

### 2.3. Measurements

For each walking gait condition, the following measurements were analyzed: footstep analysis (i.e., stride length, stride cycle, swing time, stance time) and brain activation. Footstep parameters were assessed with OptoGait (Microgait^TM^ Bolzano, Italy) on a treadmill [31] calculated from a minimum of 500 stride-to-stride cycles [29].

To analyze the variability and the stability of the cycle duration, two approaches have been used: the maximum Lyapunov exponent (LyE) within the framework of the dynamic systems theory, and the classical statistical analysis of the variability around the average cycle. The first has the advantage of probing the stability of a biomechanical system, which should not be identified with the concept of variability. Indeed, as a mechanical property, variability indicates the ability of the system to reliably perform in a variety of different environmental and task constraints, while stability refers to the dynamic ability to offset an external perturbation [32,33]. Standard statistical tools are suitable to quantify the variation in a set of data regardless of their order distribution (i.e., time ordering). Time correlations and the behavior of a biomechanical motor are effectively caught by mathematical tools, such as LyE computation, specifically developed for nonlinear systems. In this respect, the LyE evaluates the resistance of the postural control system to gait perturbations.

The maximum LyE was calculated using custom software for each subject in each walking condition. We followed the approach described in [34,35], as concerns the calculation of LyE from finite experimental dataset and the general theory of the analysis of nonlinear time series.

From the experimental time series, we defined delay-embedding vectors [34,35] for the reconstruction of the state vectors in the phase space. The autocorrelation properties of the data series were used for a first choice of the most suitable delay time, *τ*, and then the maximum LyE is evaluated as reported in [34] for different choices of the embedding dimensions *m*. We selected a “reasonable” range for *m* values from the plot (in double logarithmic scale) of the correlation sum vs. the selected minimum distance *ε* between space vectors (see 34, 35 for details), as those values for which the slope of the plot is the same on a certain *ε* interval. After that, the computation of LyE for different values of *τ* and *m* provides an average estimation (for each studied subject) and its error.

The standard deviation of cycle-to-cycle intervals (SDtot) was obtained as time domain measures to assess gait variability [23].

Electroencephalographic activity was recorded with the MindBand portable headset device [36]. The used device is equipped with active dry stainless-steel electrodes with a size of 10 mm in the elastic. The reference and ground electrodes are contained in the clip located on the ear. The electrode positions were FP1 and FP2, according to the International 10/20 System. The electric potential recorder between the active and reference electrodes was subtracted through the rejection of the common mode to generate a single signal of the EEG channel. The signal was amplified 8000 times. The EEG recording of each electrode was digitized at 128 Hz, and the data was processed in the built-in ThinkGear microchip and bandpass filtered (1–40 Hz).

The EEG signal was transmitted in wireless by Bluetooth© to a personal computer and customized software made with Origin that was able to collect each raw data. According to NeuroSky manufactory, raw EEG data were converted to voltage (raw Value × (1.8/4096))/2000 and filtered on the sampling rate to fit at 18 Hz (this is due to a 2000× gain, 4096 value range, and 1.8 V input voltage). Proprietary algorithms detect and correct eye movements and muscle artifacts in the time domain, prior to the Fast Fourier Transformation (FFT) of each epoch of 0.5 s in 0.25 Hz bands. It is possible that the artifact rejection methods are not 100% efficient, and residual artifacts may be present in the data. We have considered the low beta waves (12–15 Hz) that are commonly referred to as “Beta 1” or “Low Beta” or SMR (sensorimotor rhythm) brain waves on the sensorimotor cortex.

### 2.4. Statistical Analysis

Data were presented as means and standard deviations (±SD), calculated after verifying the normality of distributions using the Shapiro–Wilk test [37]. Data were normally distributed, so parametric statistics was used. Subsequently, an analysis of variance (ANOVA) for repeated measures with one factor (walking condition) was applied in order to determine any significant differences of stride length (cm), stride cycle (s), stance time (s), swing time (s), and beta wave signal and RPE at three different walking conditions (WS-WTS-WSI). When a significant F-value [38] was found, least-significant difference (LSD) was chosen as the post hoc procedure, while Intraclass Correlation Coefficient (ICC) was used to assess the reliability of the measures. Statistical analyses were performed using the software SPSS Statistic version 23.0 (IBM Corps., Armonk, NY, USA). The level of significance was set at *p* ≤ 0.05.

## 3. Results

**Figure 2:**
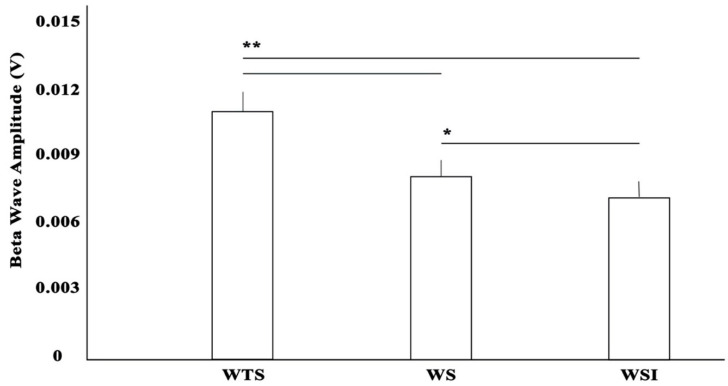
Beta wave amplitude signal in (* and ** for *p* < 0.05 and 0.01, respectively) different walking conditions (without shoes WTS, with shoes WS, with shoes and insoles WSI).

**Figure 3:**
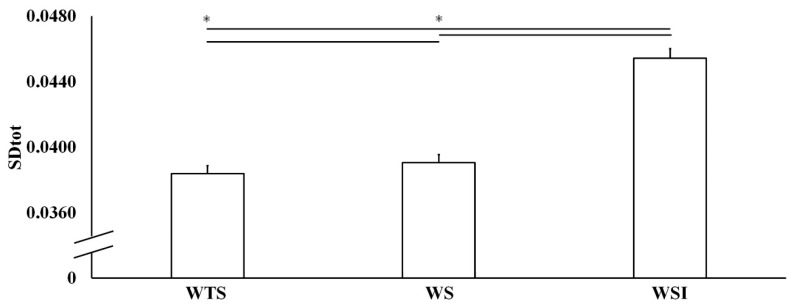
Standard deviation of cycle-to-cycle intervals (SDtot) on WTS (without shoes), WS (with shoes), WSI (with shoes and insoles). Statistical significance is denoted as “*” *p* < 0.001 between the two walking conditions.

The Shapiro–Wilk test showed a normal distribution for all measured parameters, with W values ranging from 0.942 to 0.971 (*p* > 0.05). Footstep analysis (stride length, stride cycle, swing time, stance time, Table 1) showed an excellent reliability (ICC 0.912 < 0.978). ANOVA showed F_1,10_ = 4.941 with *p* = 0.012 for the stride length, F_1,10_ = 1.734 with *p* = 0.190 for the stance time, F_1,10_ = 3.876 with *p* = 0.029 for the swing time, and F_1,10_ = 4.938 with *p* = 0.012 for the stride cycle. RPE was similar for each walking condition (RPE 1 ± 0.00; *p* > 0.05). ANOVA showed a F_1,10_ = 564.201 with *p* = 0.0001 for beta wave signal. Particularly, beta wave amplitude was higher (0.106 ± 0.001 V) in WTS compared to WS (0.0790 ± 0.0007 V) and WSI (0.0737 ± 0.0006 V), respectively, with *p* < 0.01. A small effect (*p* < 0.05) was found between WS and WSI (Figure 2). Gait variability (Figure 3) as SDtot showed a F_1,10_ = 1826.548 with *p* = 0.0001. ANOVA’s LyE showed a F_1,10_ = 3.209 with *p* = 0.056, while post hoc analysis showed a significant effect between WS and WSI with *p* = 0.023. Conversely, no significant effects were found between WTS and WS/WSI (*p* = 0.070/0.607), respectively.

On the brain activation side, during the walk, the analysis is guided by information from the nervous system. We focused our attention on the beta wave as it is particularly present in the walking state and when the subject increases his attention to solve problems. We observed that the amplitude of the beta wave was greater in the cortical and sensorimotor areas during the WTS condition compared to the WS and WSI conditions. These measurements were performed with a MindBand (MindBand, Neurosky^TM^, San Jose, CA, USA) device, a portable and low-cost device that can record a good quality EEG in proportion to its cost. Its functioning has been validated by Héctor Rieiro et al. [39] by comparing the NeuroSky with a traditional electroencephalographic system. In their work, Rieiro et al. claim that the system (NeuroSky MindWave) is limited by noise, but provides stable recordings, even for long periods of time. Furthermore, its data is of adequate quality compared to that of traditional wet electrode EEG devices, except for a potential calibration error and spectral differences at low frequencies, while the reliability (i.e., test–retest) was higher [40]. With NeuroSky, we showed that the greatest beta wave activity in the studied cortical areas was recorded in the WTS condition as a response to the greater perturbations coming from the motor sense system [41]. The lowest activity was shown by the WSI condition: we think that the insoles provide more “passive” comfort, and the foot turns off the motor sensor system; indeed, it is well documented that the use of insoles reduces muscular activity [42].

**Figure 4:**
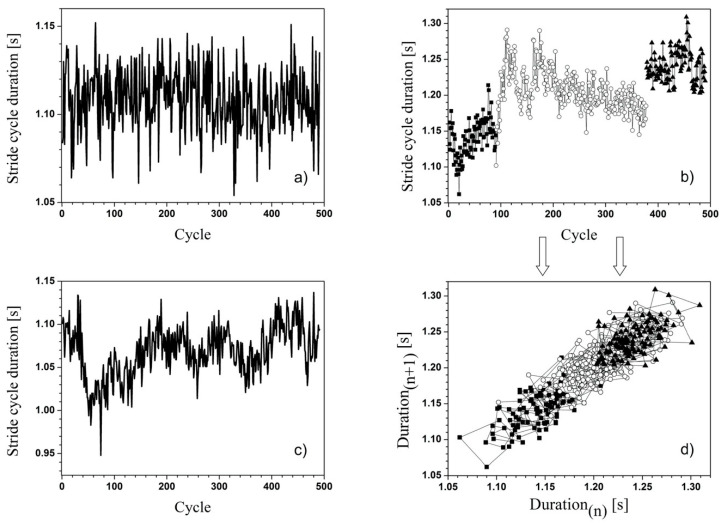
Some representative plots of the stride cycle duration during an entire walking test. All the displayed data are in WTS condition: (**a**) Very regular walking over the whole range; (**b**) a walking dataset clearly characterized by sharp “transitions” from a walking regime to another. Different regimes have been highlighted by using different point stiles (also to produce a clearer output for the plot in panel d)); (**c**) an intermediate situation between the two previous ones, characterizing most subjects; (**d**) 2D-projection of reconstructed trajectories in the phase-space produced by the data of panel b): the different symbols highlight the presence of local attractors for the orbits.

The plots of the experimental time-series for the duration of stride cycles revealed some qualitative differences among the subjects. Figure 4 shows some examples of time behavior of the walking subjects. There were extremely regular behaviors (Figure 4a), in which any time range was basically undistinguishable from the others; therefore, they were characterized by a relatively low global variability of the dataset. On the contrary, some subjects exhibited a walking behavior characterized by “sudden” jumps from a range of gait cycle duration values to another, as in Figure 4b (it is worth noting that in such a case, a simple SD analysis would not be able to differentiate such a behavior from another one with the same values, but homogeneously distributed along the duration of the walking experiment). Finally, some intermediate behaviors were detected (see Figure 4c).

The analysis of autocorrelation functions, as well as the 2- and 3-dimensional projections of the reconstructed trajectories in the phase-space, revealed that the observed fluctuations in the time series were not merely noise, but had a structure and an order that deserved investigation with appropriate tools. Namely, the numerically calculated autocorrelation functions were not flat, and revealed correlation properties over ranges of many cycles (of the order of 100 cycles for most of recorded dataset) even for behaviors far from the evident time-order of Figure 4b. In the same way, the 2D-projection of reconstructed trajectories clearly showed, for most datasets, the presence of attractors in the phase-space, proving local stability and order of the time-series (an example is reported in Figure 4d, inferred from the plot of Figure 4b, and highlighted by representing the points in different gait ranges with different symbols; it must be always taken into account that such a 2D-plot is only a two-dimensional projection of an orbit in a multidimensional space, thus producing overlapping nodes not really present in the original orbit).

**Figure 5:**
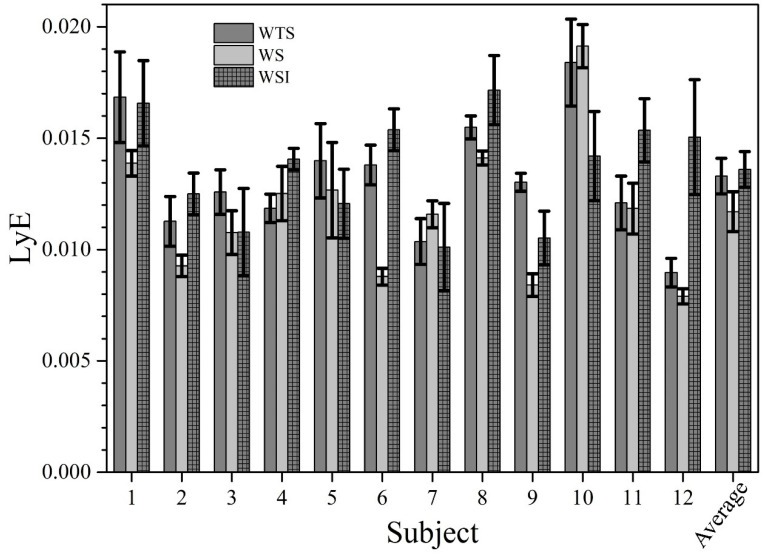
Maximum Lyapunov exponents (LyEs) calculated for each subject in each investigated walking condition. LyE values are measured in (stride cycles) ^−1^.

Once the preliminary analysis had clarified the nonstochastic nature of the measured fluctuations, we proceeded with the numerical computation of LyEs for each investigated subject in each of the three investigated conditions. The results are summarized in Figure 5, in which LyEs are reported as a function of the subject for each walking condition.

## 4. Discussion

The aim of this study was to assess the walking gait variability and stability of different tasks related to brain activity without shoes (WTS), with shoes without insoles (WS), and with shoes and preformed insoles (WSI). We found that the stride length in the WSI condition increased compared to the WTS and WS conditions (4% and 1%, *p* < 0.05). The increase of stride length can be justified with the Oeffinger study [10], i.e., a pendulum lengthening effect caused by the extra weight of the shoes. In this frame, we can explain the higher stride length in the WSI condition compared to WS in terms of the extra weight of the preformed ergonomic insoles inside the shoes.

In addition, the increased stride length in the WSI condition could be explained by the cushioning effect created by the insoles and a possible better reuse of elastic energy or a better use of muscle energy during the push-off phase. These hypotheses should be tested with other more specific studies. Obviously, we have the opposite behavior of the stride frequency, determined here as the inverse of stride cycle time; in fact, the lowest value was observed in the WTS condition. Regarding the kinematic analysis, our results are consistent with previous literature comparing barefoot and shod walking conditions [3].

The most important finding of this study is the modification of the walking gait variability using SDtot (Figure 3) and stability (Figure 4 and Figure 5) using the LyE according to previous studies [23]. Estimated LyEs (Figure 5) exhibit a certain value distribution, reflecting the above-mentioned qualitative differences among subjects. LyE is introduced inside the frame of nonlinear dynamics, and it measures the divergence from an intended trajectory in the phase-space after a small perturbation (basically, the LyE is the inverse of the divergence time: smaller LyE corresponds to larger divergence time and, therefore, to smaller divergence rate, i.e., to greater stability of trajectories in the phase-space of the system). For the experimental dataset, compared to analytically calculated time-evolutions, the challenge is to infer LyE from a finite, unidimensional, time-series of data also affected by noise, which represents an additional difficulty in performing reliable computation. However, it seems that the WS condition was characterized by LyE values consistent with or lower than those in WTS and WS conditions; this observation applied to all subjects, with only one remarkable exception (discussed below). Besides the observation of the LyE plots (Figure 5), this circumstance was also confirmed by simply calculating the averages/SD of the estimated LyE values in the three conditions: 0.0133 ± 0.0008, 0.0117 ± 0.0009, and 0.0136 ± 0.0008 for WTS, WS, and WSI, respectively.

Our interpretation is that the shoes, compared to the barefoot condition, favor the capability to offset the effects of possible external perturbations, guaranteeing improved biomechanical stability (lower LyE means lower divergence rate of the orbits in the phase-space). It is worth nothing that the only clear exception to this general trend is the subject (number 10 in Figure 5) characterized by the greatest regularity (displayed in Figure 4a). Although further statistical analysis would help to clarify the connections between these two features, from our point of view, it is very interesting how the shod condition actually perturbed the subjects, but the addition of anatomical insole leads to walking parameters more similar to the barefoot conditions.

In this frame, it could appear strange that the addition of insoles seems to lead to LyE values greater on average than those with only shoes. We consider it reasonable to exclude that this could be related to a mere loosening of biomechanical stability, at the least as concerns global stability, defined operationally as the probability of falling [43]. More likely, the LyE analysis quantifies the response to small (local) perturbations, and our findings prove that the application of insoles restores, under this point of view, a behavior similar to that of barefoot walking, confirming that the use of insoles could be effective in making the inside of the shoes more anatomical and ergonomic. Compared to the WS condition, the LyE result for WSI walking can be also viewed in terms of stride length (greater in WSI), perceived exertion (same), and brain activation (lower). Indeed, the lower brain activation (without any larger perceived exertion) corresponds to a lower cognitive engagement, and could simply be due to lower reaction to external perturbations, which should not be viewed as a negative effect. Under a different point of view, in this situation the subject “does not need” to offset the effect of perturbations, being able to walk without efforts, i.e., comfortably, at an increased stride length, and therefore switching to a more passive control of muscle (indicated by the lower brain activation). It is also plausible to speculate that the WS condition is the “normality” for the subjects because they usually use shoes during the day: the larger stride length might indicate a new movement pattern that the neuromuscular system is not in the habit of (a preliminary habituation to the WSI condition could decrease the effect of such potential bias). In addition, the increased stride length can be a source of great time variability for the gait cycles. However, our results do not seem to support the evidence of a detrimental effect of shoes, compared to barefoot condition, in gait stability and walking comfort.

## 5. Conclusions

Further studies in this way should be performed in order to better understand these results and to create normative data to compare measurements of different populations and disease states.

From the cerebral point of view, we have shown that during the walk, a reduction of the ground stimulation by shoes with (WSI condition) or without insoles (WS condition) corresponds to a reduction in the amplitude of the beta wave, known to be implicated in the processes of attention. In fact, the amplitude of the beta wave was greater when walking without shoes, where the foot is bare and its nerve receptors are stimulated by contact with the ground, compared to the situation in which the feet are closed in shoes that reduce the stimulation of the nervous receptors. From the kinematic point of view, the gait variability is lower with shoes (WS condition) because it is the subjects’ usual way to walk, but the behavior without shoes (WTS) and with shoes and insoles (WSI) is relatively similar. The results of the study refer to an acute condition; further studies are needed to understand human adaptation to chronic WTS and WSI conditions.

## Figures and Tables

**Table 1:** Footstep data of the three different walking conditions.

Variables	WTS	WS	WSI	WTS-CI	WS-CI	WSI-CI
Stride length (cm)	61.59 ± 2.05 *^,§^	63.38 ± 2.05 ^†^	64.09 ± 2.40	60.40–62.77	62.20–64.57	62.71–65.48
Stride cycle (s)	1.11 ± 0.04 *^,§^	1.14 ± 0.04	1.15 ± 0.04	1.089–1.128	1.119–1.159	1.128–1.174
Stance time (s)	0.799 ± 0.033	0.817 ± 0.036	0.824 ± 0.041	0.779–0.818	0.796–0.838	0.800–0.847
Swing time (s)	0.309 ± 0.016 ^§^	0.321 ± 0.016	0.326 ± 0.017	0.300–0.319	0.312–0.330	0.317–0.324

Note: data are expressed as mean ± SD, 95% Confidence Interval (CI) for three walking gait conditions: with shoes (WS), without shoes (WTS), with shoes and insoles (WSI). Significance differences (*p* < 0.05) as post hoc analysis was showed for “*”—WTS/WS “^§^”—WTS/WSI “^†^”—WS/WSI
